# Supplementary material for: Mental and physical health in persons receiving inpatient pulmonary rehabilitation treatment for post-COVID condition
Source: PLoS One. 2025 Aug 26;20(8):e0330938. doi: 10.1371/journal.pone.0330938 (PMC12380288; doi:10.1371/journal.pone.0330938)
Supplement: Attachment — Submitted filename: comments.docx [file pone.0330938.s002.docx]

Editor’s comments:

1. What were the inclusion/exclusion criteria for the sample?

*RESPONSE: There were no inclusion or exclusion criteria other than that patients were admitted for post-COVID condition, as outlined in the sample paragraph of the method section.*

2. What were the participants’ pre-COVID mental health conditions?

*RESPONSE: We have now added self-reported mental disorders before the first COVID infection to Table 1.*

3. What scoring systems have been validated or are they widely used in COVID-related research?

*RESPONSE: All of the used measures are widely used in COVID-related research (e.g.,* [*https://doi.org/10.1186/s12912-021-00800-2*](https://doi.org/10.1186/s12912-021-00800-2)*,* [*https://doi.org/10.12688/f1000research.50781.1*](https://doi.org/10.12688/f1000research.50781.1)*,* [*https://doi.org/10.2147/PRBM.S329380*](https://doi.org/10.2147/PRBM.S329380)*,* [*https://doi.org/10.1186/s12905-024-03102-2*](https://doi.org/10.1186/s12905-024-03102-2)*,* [*https://doi.org/10.3389/fpsyt.2021.695678*](https://doi.org/10.3389/fpsyt.2021.695678)*,* [*https://doi.org/10.23937/2572-4037.1510049*](https://doi.org/10.23937/2572-4037.1510049)*,* [*https://doi.org/10.1080/21641846.2023.2295419*](https://doi.org/10.1080/21641846.2023.2295419)*,* [*https://doi.org/10.1371/journal.pone.0293081*](https://doi.org/10.1371/journal.pone.0293081)*,* [*https://doi.org/10.1007/s12144-022-02833-5*](https://doi.org/10.1007/s12144-022-02833-5)*). However, we have restricted to citing only the most relevant studies in the discussion section.*

4. What was the correlation between physical health with changes in mental health.

*RESPONSE: We are not sure how to answer this question. Pulmonary functioning variables were only available at admission, as described in the method section. While there is one subscale of the SF-36 entitled “physical functioning”, the other subscales of the SF-36 as well as the PHQ-D also assess aspects of physical health or aspects for which physical and mental health cannot be clearly separated (e.g., somatoform syndrome/somatic symptoms severity, energy/fatigue, pain). Thus, we are not sure which correlations the editor is interested in but we would be happy to address this in another revision if the editor can clarify this.*

Reviewer’s comments:

Abstract:

1. I would suggest including length of treatment in the abstract.

*RESPONSE: We have now added mean length of treatment to the abstract.*

Introduction:

2. The introduction could be improved by more clearly articulating the study rationale and research gap addressed.

*RESPONSE: We have now expanded on this by explicitly describing three study aims and their rationale in the final paragraph of the introduction section.*

3. It would also be strengthened by providing justification for focusing on pulmonary rehabilitation, specifically in relation to its potential impact on mental health.

*RESPONSE: See response above.*

4. I would suggest discussing the bidirectional relationship between physical and mental health impairments to provide a stronger theoretical framework for the study.

*RESPONSE: See response above.*

Methods:

5. Were there inclusion/exclusion criteria for the sample?

*RESPONSE: There were no inclusion or exclusion criteria other than that patients were admitted for post-COVID condition, as outlined in the sample paragraph of the method section.*

6. Are data on participants’ pre-COVID mental health conditions available?

*RESPONSE: Yes, we have now added self-reported mental disorders before the first COVID infection to Table 1.*

7. Are data on the time between COVID-19 infection and admission to rehabilitation available?

*RESPONSE: We have now added to the sample description that “time between the first COVID infection and admission to the hospital approximately ranged between 1–3 years (as the exact date of the first COVID infection could not be exactly determined retrospectively, no precise numbers can be reported here).”*

8. Have these measures been validated or widely used in COVID-related research?

*RESPONSE: Yes, the PHQ-9/PHQ-15, PC-PTSD, and SF-36 are widely used in COVID-related research (e.g.,* [*https://doi.org/10.1186/s12912-021-00800-2*](https://doi.org/10.1186/s12912-021-00800-2)*,* [*https://doi.org/10.12688/f1000research.50781.1*](https://doi.org/10.12688/f1000research.50781.1)*,* [*https://doi.org/10.2147/PRBM.S329380*](https://doi.org/10.2147/PRBM.S329380)*,* [*https://doi.org/10.1186/s12905-024-03102-2*](https://doi.org/10.1186/s12905-024-03102-2)*,* [*https://doi.org/10.3389/fpsyt.2021.695678*](https://doi.org/10.3389/fpsyt.2021.695678)*,* [*https://doi.org/10.23937/2572-4037.1510049*](https://doi.org/10.23937/2572-4037.1510049)*,* [*https://doi.org/10.1080/21641846.2023.2295419*](https://doi.org/10.1080/21641846.2023.2295419)*,* [*https://doi.org/10.1371/journal.pone.0293081*](https://doi.org/10.1371/journal.pone.0293081)*,* [*https://doi.org/10.1007/s12144-022-02833-5*](https://doi.org/10.1007/s12144-022-02833-5)*). However, we have restricted to citing only the most relevant studies in the discussion section.*

9. Did changes in physical health during treatment corresponded with changes in mental health?

*RESPONSE: We are not sure how to answer this question. Pulmonary functioning variables were only available at admission, as described in the method section. While there is one subscale of the SF-36 entitled “physical functioning”, the other subscales of the SF-36 as well as the PHQ-D also assess aspects of physical health or aspects for which physical and mental health cannot be clearly separated (e.g., somatoform syndrome/somatic symptoms severity, energy/fatigue, pain). Thus, we are not sure which correlations the reviewer is interested in but we would be happy to address this in another revision if the reviewer can clarify this.*

10. Did duration of treatment predict any outcomes?

*RESPONSE: It did not, which we now report in Footnote 1.*

Discussion:

11. The introduction emphasizes the need to evaluate treatments for post-COVID condition, but the discussion does not fully tie findings back to treatment relevance.

*RESPONSE: We agree and have now restructured the discussion section (e.g., introduced subheadings) and have added more discussion about treatment effects.*

12. The discussion includes extensive comparisons to prevalence rates and scores shown in previous studies, which makes it challenging to discern the central takeaways from the current study. I would recommend streamlining comparisons to previous studies and focusing Focus on contextualizing the findings within the broader literature without overwhelming the reader with extensive numerical comparisons.

*RESPONSE: We agree and have now restructured and rewritten the first few paragraphs of the discussion section, for example, by removing the detailed numbers found in other studies and instead describe more broadly how the current findings compare to other studies.*

13. The authors might say more about somatoform syndrome, as it may be less familiar to readers compared to anxiety and depression.

*RESPONSE: We have now added to the description of the PHQ in the methods section that this refers to a pattern of multiple, recurrent, and frequently changing physical symptoms.*

14. I would suggest discussing the clinical and public health implications of the findings more thoroughly, especially in terms of rehabilitation and mental health care for post-COVID condition.

*RESPONSE: We have now expanded on this in the discussion section by discussing that, as inpatient treatment is more expensive than daypatient or outpatient treatment, future studies are needed that compare different treatment settings in terms of cost-benefit efficacy.*

15. The discussion could be strengthened by highlighting what is novel and unique about this study.

*RESPONSE: One new aspect is that we report on changes in self-reported mental and physical problems during a specialized inpatient pulmonary rehabilitation treatment. However, there are, of course, other similar studies und parts of the current manuscript report on findings that have been investigated in other studies as well. PLOS One’s policy is that novelty and uniqueness are no prerequisites for publication of scientific studies (*[*https://everyone.plos.org/about-plos-one*](https://everyone.plos.org/about-plos-one)*), similar to suggestions by others (*[*https://doi.org/10.3389/fpsyg.2021.609802*](https://doi.org/10.3389/fpsyg.2021.609802)*). Thus, we feel that highlighting novelty and uniqueness is not required or may even be inappropriate in light of the journal’s policy.*

16. While self-report bias is mentioned as a limitation, its potential impact on the study’s findings is not discussed.

*RESPONSE: We have now extended this sentence such that potential self-report biases may possibly lead to an overestimation of the prevalence of psychosomatic and psychological conditions or treatment effects.*
