# Supplementary material for: Mental and physical health in persons receiving inpatient pulmonary rehabilitation treatment for post-COVID condition
Source: PLoS One. 2025 Aug 26;20(8):e0330938. doi: 10.1371/journal.pone.0330938 (PMC12380288; doi:10.1371/journal.pone.0330938)
Supplement: Attachment — Submitted filename: comments_auresp_2.docx [file pone.0330938.s003.docx]

Reviewer #2:

1. Clarify the clinical significance of symptom reduction, especially for PTSD and anxiety. A one-sentence addition noting whether changes meet known thresholds for clinical relevance would help contextualize the results for clinicians.

*RESPONSE: We agree have reformulated and expanded on this in the “Changes from admission to discharge” section in the discussion section: “Somatic and depressive symptoms severity, generalized anxiety, and stress as assessed with the PHQ significantly decreased from admission to discharge with small-to-large effect sizes. In contrast, symptoms of panic disorder, eating disorders, and PTSD did not change from admission to discharge, suggesting that patients with post-COVID condition presenting with these symptoms require specialized psychotherapy in addition to pulmonary rehabilitation treatment.”*

2. In Table 1, consider highlighting the mental health scales where improvement was statistically significant. Asterisking or bolding these values can guide readers better.

*RESPONSE: Thank you for this suggestion. We believe the comment may refer to Table 2, as Table 1 presents only descriptive statistics. In Table 2, we have indeed included asterisks to indicate statistically significant correlation coefficients, and we fully agree that this enhances the table’s readability. Regarding the results on changes from admission to discharge, we reported the exact p-values in the table. For that reason, we decided not to use asterisks, as we felt they would be redundant in this context. For example, annotating a value with both “< 0.001” and a table note stating “p < .005” does not provide additional clarity, as the exact p-value already conveys that the result is statistically significant.* *We hope this explanation clarifies our approach, and we’re happy to adjust the presentation if further clarification is needed.*

3. The addition of the correlation between pulmonary function and self-reported physical health is appreciated. Still, a short note in the Discussion section interpreting the lack of correlation between pulmonary function and mental health outcomes would add nuance to the findings.

*RESPONSE: We agree and have added this sentence to the “Relationships between pulmonary functioning and self-report measures” section in the discussion section: “Thus, it appears that while persons with post-COVID condition report high rates of mental health issues, these do not seem to be directly attributable to impaired pulmonary functioning.”*

4. Typographical Clean-Up: One or two sentences could benefit from slight tightening. Example: "Mental and physical problems partially remit..." could be refined to "Both mental and physical symptoms showed partial improvement

*RESPONSE: We have now reformulated these sentences as suggested.*

Reviewer #3:

Previous Comment:

1. In extension to editor's comment 1. What were the inclusion/exclusion criteria for the sample? The answer could be all-comers, if there is no inclusion exclusion criteria.

*RESPONSE:* *We have now reformulated this sentence as follows to be more precise: “Data of a consecutive sample of 141 persons who were infected with COVID–19 between 2020 and 2023 and who were admitted to inpatient pulmonary rehabilitation treatment for post-COVID condition at the Schön Klinik Berchtesgadener Land (Schönau am Königssee, Germany) between 16/01/2023 and 16/12/2023 were accessed on 19/06/2024 and analyzed. No additional inclusion or exclusion criteria were applied.”*

New comments:

1. Please include severity of Covid infection, length of treatment, and ICU admission of patients at the time of COVID infection and in result section severity comparison on study results.

*RESPONSE: Thank you for this comment. We would like to clarify that severity of COVID-19 infection—based on the WHO Ordinal Scale for Clinical Improvement—and whether patients were hospitalized is already presented in Table 1. The length of pulmonary rehabilitation treatment is also provided there. If the reviewer is referring instead to the duration of acute COVID-19 treatment at the time of initial infection, unfortunately, we do not have access to that information in our dataset. Regarding ICU admission, as shown in Table 1, the vast majority of patients (87%) received ambulatory care and were not hospitalized (data were missing for 4 individuals). Because of this strong imbalance—e.g., only 18 patients were hospitalized—it was not feasible to conduct meaningful comparisons in the inferential analyses. We hope this clarification is helpful and are happy to revise further if the reviewer had something different in mind.*

2. It is suggested to follow standard numerical presentation throughout the manuscript. e.g. in Measures section .81 should be replaced with 0.81.

*RESPONSE: We have now added the leading zeros to the decimal numbers for which we had previously omitted the leading zeros (i.e., decimal numbers that cannot exceed -1 or 1, cf. Publication Manual of the American Psychological Association 7^th^ edition).*

3. Elaborate pulmonary rehabilitation treatment to patients in includes what type of treatment was given and length of treatment.

*RESPONSE: Thank you for this comment. We would like to point out that details on the pulmonary rehabilitation treatment are already included in the manuscript. Specifically, the “sample” section (first paragraph of the Methods) describes the key treatment components—namely diagnostic assessments, medical treatment, endurance and strength training, patient education, respiratory physiotherapy, relaxation techniques, occupational therapy, psychological support, and nutrition counseling (Gloeckl et al., 2021). Additionally, the length of treatment is reported in Table 1. We hope this addresses the reviewer’s suggestion, and we are happy to further clarify or elaborate if needed.*

4. Present manuscript is a small sample size but it mentioned Because of the numerous inferential tests and large sample size, we considered effects as significant at p < .005 (cf. Benjamin et al., 2018). Author needs to clarify how and why redefined p value 0.005 is applicable in the line of cited reference.

*RESPONSE: Thank you for raising this important point. We have reworded the sentence in the manuscript to read: “Because of the numerous inferential tests and large sample size, we considered effects as significant at p < 0.005, as has been recommended by Benjamin et al. (2018), who argue that this alpha level ‘represents “substantial” to “strong” evidence according to conventional Bayes factor classifications’ and ‘would reduce the false positive rate to levels we judge to be reasonable’ (p. 7).” We understand the reviewer’s concern regarding the characterization of the sample size. While it may be considered modest in absolute terms, we believe it is sufficiently large for the types of analyses conducted. Specifically, the sample provided ample statistical power to detect even small effect sizes in both between-person correlations and within-person pre-post comparisons. In these contexts, effects such as correlations below r = 0.3 and Cohen’s d below 0.5 reached significance at the adjusted alpha level of 0.005. We hope this clarification helps justify the chosen significance threshold, and we remain open to further adjustment or elaboration if needed.*

5. It has been 1.5 years since last follow-up and linger follow-up and comparison to baseline would be helpful.

*RESPONSE: Thank you for this valuable suggestion. We would be happy to clarify that although 1.5 years have passed since data collection was completed, collecting additional follow-up data is unfortunately not feasible. Specifically, our ethical approval did not include provisions for recontacting participants, and the data have been fully and irreversibly anonymized in accordance with those ethical standards. As such, while we agree that longer-term follow-up would indeed offer meaningful insights, this lies beyond the scope of the current study’s approved framework.*

6. Please provide the role of each author in present manuscript.

*RESPONSE: We had actually already indicated the author contributions in the submission system but have now additionally included these in the manuscript.*
